# Understanding How Rurality Relates to Residents' Experiences of Accessing Primary Care: An Interview‐Based Study

**DOI:** 10.1111/ajr.70153

**Published:** 2026-02-09

**Authors:** Maddie Higgins, Tiana Gurney, Matthew McGrail

**Affiliations:** ^1^ Rural Clinical School The University of Queensland Brisbane Queensland Australia

**Keywords:** Australia, geographic fragility, health equity, primary care access, qualitative research, rural health

## Abstract

**Objective:**

To explore the experience of local residents when accessing primary care across different levels of rurality.

**Setting:**

Four discrete regional, rural, and remote communities in Queensland, Australia, categorised by the Modified Monash Model classification.

**Participants:**

Residents from a regional centre (*n* = 15), a small rural town (*n* = 9), and two remote or very remote communities (*n* = 6).

**Design:**

Semi‐structured interviews using thematic analysis with both deductive and inductive coding.

**Results:**

Common challenges across all levels of rurality included limited primary care provider availability, long wait times, and disrupted care continuity, though their impact varied by the level of rurality. Regional centre residents predominantly experienced availability and timeliness challenges, while the small rural town residents faced availability, timeliness, and geography challenges. The remote and very remote communities experienced interconnected challenges, including a lack of permanent primary care providers, extensive travel, disrupted care continuity, and poor understanding of the community culture by some primary care providers. A key finding was ‘geographic fragility,’ which increased with the level of rurality, where remote community residents had to weigh up whether their health needs justified the burden of accessing primary care.

**Conclusion:**

This study highlights the need for nuanced, context‐specific approaches for improving primary care access across different levels of rurality, with particular attention to geographic fragility in remote and very remote communities. Policy implications include developing targeted workforce strategies, addressing timeliness challenges, and implementing flexible service models to ensure equitable access to primary care for all Australians.

## Summary

- What is already known on this subject?
  ○ Adequate access to primary care is vital for a strong health system, though deficiencies persist for Australia's rural and remote communities.
  ○ Healthcare access is multidimensional, of both barriers and enablers.
  ○ Research has explored single dimensions of access, though a comprehensive exploration of these across different levels of rurality is lacking.
- What does this study add?
  ○ A nuanced understanding of the dimensions of access when accessing primary care across regional, rural, and remote Australian communities.
  ○ Demonstration of how perceptions of primary care access vary across different levels of rurality, highlighting the need for contextually tailored approaches to policy design.
  ○ Identifies the concept of geographic fragility in smaller and isolated communities, providing novel insights into rural and remote primary care access.

## 1 Introduction

Australian residents outside of metropolitan areas (around 30% of Australia's population) [1] are known to have poorer health, which is related to poorer access to healthcare and higher rates of socioeconomic disadvantage, as well as reduced levels of employment, education, and income [1, 2]. Differences in health behaviours and outcomes are influenced by diverse experiences including individual, social, and environmental factors. The healthcare workforce is continually evolving to respond to these diverse and complex care needs [1]. Addressing healthcare access challenges for rural and remote residents, particularly in accessing primary care for health system entry and non‐urgent but critical ongoing healthcare, remains a persistent focus for policymakers.

Primary care forms the foundation of an effective healthcare system [3] and serves as the first point of contact for most health‐related concerns. Primary care is largely delivered by general practitioners (GPs) in combination with other healthcare providers including nurses, pharmacists, and allied health workers [4]. In addition to treating both acute and chronic conditions, primary care providers focus on preventive measures, health promotion, and managing common health issues such as routine check‐ups, vaccinations, and disease management [5]. Increasing demands on primary care can make accessing healthcare challenging during times of need. Accessing primary care is often a complex and increasingly difficult experience for residents of regional, rural, and remote Australian communities [6, 7].

Access challenges to primary care in regional, rural, and remote Australian communities are multifaceted. Workforce shortages are a persistent challenge, with many regional, rural, and remote communities struggling to attract and retain sufficient primary care providers [8, 9, 10, 11, 12]. Geographic isolation compounds this problem, as vast distances between communities and primary care services can make routine visits logistically challenging and time‐consuming [8, 10, 11, 12, 13, 14, 15]. Additionally, costs relating to accessing primary care services, including direct and indirect costs such as travel and time off work, can pose significant financial challenges for many individuals and families [10, 11, 15]. These challenges, however, represent only some of the access difficulties and are not necessarily consistent across different levels of rurality. The experiences of accessing primary care for those living in regional centres may differ from those in small rural towns or remote and very remote communities.

The key dimensions of healthcare access have been broadly studied over several decades and summarised in different models and frameworks [16, 17, 18, 19]. Many of which continue to inform research and policy development. This evidence largely derives from metropolitan contexts characterised by adequate access to healthcare services. One exception is Russell and colleagues' rural‐focused study, which identified seven dimensions of access to primary care, including availability, geography, affordability, accommodation, timeliness, acceptability, and awareness [20]. More recently, research has assessed how these seven dimensions of access varied in their importance across different levels of rurality when accessing primary care [21]. There is a need for a richer understanding of how rurality relates to residents' experiences of accessing primary care.

This study aims to explore local residents' lived experiences of key challenges when accessing primary care in regional, rural, and remote communities in Queensland, Australia. By exploring lived experiences, it seeks to identify how dimensions of access are perceived and experienced across different levels of rurality. This nuanced understanding is essential for informing policy interventions and innovative solutions that can effectively address the challenges faced by residents in different rural and remote communities.

## 2 Method

### 2.1 Design and Setting

This study employed a qualitative descriptive design to explore lived experiences of accessing primary care [22]. Discrete regional, rural, and remote Australian communities, defined by the Modified Monash Model classification, were chosen to capture and contrast residents' contextual experiences. Four communities in rural and remote Queensland were selected for the study, including a regional centre, a small rural town, a remote community and a very remote community (the latter two combined as ‘remote’). Each community had access to at least one local primary care service with an in‐person general practitioner, some part of the week, and was not classified as being ‘nearby’ (< 15 min travel) to any large urban communities. A low‐risk ethics application was approved for this study. The methodology and results are reported following the consolidated criteria for reporting qualitative research (COREQ) [23].

### 2.2 Recruitment

Initially, a survey was developed to collect primary data about access experiences from residents in these communities. Participants were provided with detailed information about the study's purpose and methods before giving their informed consent to complete the survey. Further details of the recruitment sampling, invitation methods and options for completing the survey are published elsewhere [21]. Upon survey completion, participants were provided with the opportunity to consent to participate in a subsequent interview, arranged at a later date. Interview participants were selected from those who expressed interest in a follow‐up interview, using a convenience sampling approach.

### 2.3 Data Collection

All semi‐structured interviews were conducted by the lead author. Not all who volunteered their interest in being interviewed were subsequently chosen. The sample size was determined based on information power, where recruitment continued until sufficient rich and detailed data had been collected to address the research aim and generate strong thematic insights [24]. The interview guide, shown in Table 1, was developed around the seven dimensions of access identified in the Russell framework [20]. The interview guide was piloted with two participants to test the clarity of interview questions. Feedback from the pilot informed minor adjustments to the wording of questions before data collection.

**TABLE 1:** Interview guide.

Question	Specific prompts	General prompts
Could you start by telling me a little about yourself? Things like your location, how often you visit a GP, and your general health?	How does this affect you? b. Do you have an example of this?	Could you please expand on that? That is very interesting, could you tell me more? Really, what was that like? Reflecting on ____________________, could you give me a bit more detail about your experience with ____________________?
Are the volume and type of services offered by your GP clinic a positive or negative aspect when visiting a GP?		
2 Is the proximity of your GP to your home a positive or negative aspect when visiting a GP?		
3 Are the direct or indirect costs a positive or negative aspect when visiting a GP?		
4 Is booking and attending a GP appointment a positive or negative aspect when visiting a GP?		
5 Is the time it takes for health care to be provided by your GP clinic a positive or negative aspect when visiting a GP?		
6 Are attitudes about cultural and social beliefs a positive or negative aspect when visiting a GP?		
7 Is the communication around your health care needs with your GP a positive or negative aspect when visiting a GP?		

Interviews were conducted via Zoom or telephone, depending on the participant's preference and geographic location. All interviews were audio‐recorded with the participant's consent and later transcribed verbatim for analysis by a professional transcription service. All transcripts were subsequently checked for accuracy by the lead author. To maintain confidentiality, all identifying information was removed from the transcripts, and participants were assigned a code.

### 2.4 Data Analysis

Thematic analysis was employed to analyse the interview data, using Microsoft Excel to facilitate data management and coding. Following Braun and Clarke's six‐phase approach, the analysis process included a combination of both deductive and inductive coding [25]. For the deductive analysis, themes were guided by Russell's seven dimensions of access [20]. For the inductive analysis, additional themes and sub‐themes were identified as they emerged from the data. The analysis process involved familiarisation with the data through repeated reading of the transcripts, generation of initial codes, searching for themes, reviewing and refining themes, defining and naming themes and producing the report [25]. All authors were involved in the coding process to ensure accuracy and trustworthiness. Any discrepancies in coding were discussed and resolved through consensus.

### 2.5 Reflexivity and Trustworthiness

The research team engaged in regular debriefing sessions to discuss interpretations and provide their perspectives on the data. The researchers acknowledged their positions within the study, requiring conscious effort to avoid preconceived views regarding participant experiences. The research team included academics with extensive expertise in conducting rural health research, using both quantitative and qualitative research methods; all have lived and worked in rural communities for > 30 years, though none work directly in primary care. Detailed records were maintained throughout the analytical process to document decision‐making and theme development.

## 3 Results

A total of 30 semi‐structured interviews were conducted with participants from a regional centre (*n* = 15), a small rural town (*n* = 9), and a remote and very remote community (*n* = 6). Overall, the sample was skewed towards females, with participants most commonly aged between 55 and 74 years, and most participants reported good health, which may have influenced perspectives on access to primary care across different levels of rurality. A full breakdown of participant demographics is presented in Table 2. Results are summarised for each access dimension.

**TABLE 2:** Participant demographics.

Characteristics	Total *n* (%)
*Rurality*	
Regional centre	15 (50)
Small rural town	9 (30)
Remote communities	6 (20)
*Age range*	
18–34	7 (24)
35–54	4 (13)
55–74	15 (50)
75 and over	4 (13)
*Sex*	
Male	10 (33)
Female	20 (67)
*Annual gross household income*	
$0–$30 000	7 (23)
$30 001–$60 000	8 (27)
$60 001–$90 000	6 (20)
$90 001–$120 000	4 (13)
$120 000 and over	5 (17)
*General health*	
Poor	2 (7)
Fair	8 (27)
Good	13 (43)
Excellent	7 (23)
*GP visits per year*	
0–1	2 (7)
2–5	12 (40)
6–12	11 (37)
More than 12	5 (16)

### 3.1 Availability

Workforce shortages emerged as a challenge across all levels of rurality. Regional centre participants experienced shortages which created appointment competition: *There are just not enough medical practitioners around … to be able to deal with the workload* (Regional centre resident 003). Complex health conditions intensified anxiety about primary care provider retention: *I'm quite concerned at the idea that I would have to find somebody else … because I have some very complex conditions* (Regional centre participant 014).

Small rural town participants faced challenges with population growth in the community overwhelming limited primary care services, leading to practice closures and community anxiety: *Initially, because the area of [small rural town] and surrounds is expanding quite largely, a couple of GP surgeries have had to close … they were finding it very difficult to find doctors* (Small rural town participant 004).

Participants from the remote communities experienced the most prominent challenges with primary care provider shortages: *We have one GP in our community, although sometimes, when they take leave or they're away … we don't have access to a GP* (Remote community participant 001).

Some concerning primary care gaps emerged: *It was three weeks after that [initial appointment] someone from the surgery rang me to say, did you know that there's results here for you? Because there had been no doctor to review them* (Remote community participant 001).

‘Closed books’ created access barriers despite the presence of primary care services: *Well, the stuff with the closing down of the practice was quite stressful because all of the other GPs in town were fully booked out and weren't taking new patients* (Small rural town participant 003).

Primary care provider instability was interconnected in the remote communities by a lack of understanding of the community culture: *Some locums have come that aren't from our [remote] community, so they don't quite grasp the nature of how far away you live, that you can't get to a pharmacy on the weekend* (Remote community participant 001).

### 3.2 Geography

Geographic challenges intensified proportionally with the level of rurality. The regional centre offered primary care services within close proximity: *The distance is not great, so it doesn't take me long to get to the GP … probably only takes me about 10–15 min* (Regional centre participant 009), though thresholds were evident: *If it was more than 20 min' drive, most of the time I probably wouldn't bother going* (Regional centre participant 011).

Small rural town participants experienced minimal challenges when primary care services were available in the town centre: *It's only* a *couple of minutes drive from our place … I guess it just makes it easy for us to access both from home and from work and from the kids' school and day‐care* (Small rural town participant 003) but faced challenges when travel was required to access primary care: *[GP] is 40 min away. I mean, obviously you have to have a car to get there because there's no public transport* (Small rural town participant 006).

Participants from the remote communities faced extensive spatial access challenges: *We do travel to [regional centre], which is almost 250 km from home, just to see a GP* (Remote community participant 004), altering primary care‐seeking behaviour: *You have to make that decision, is it going to be worth it—the travel time* (Remote community participant 001).

Access to transportation was a necessity as the level of rurality increased. Small rural town participants demonstrated this dependency: *My husband is able to drive, but I have friends that don't drive and they don't really have a choice. They need to be near a doctor* (Small rural town participant 007), creating primary care access challenges in the remote communities: *I'm physically capable of driving … other people in the community are not … which means that while I have a choice, they don't* (Remote community participant 002).

### 3.3 Affordability

Bulk billing enabled better access to primary care. Families strategically managed access: *If it's a tough week or coming to the end of our pay cycle, then sometimes it just has to wait. Whereas with the kids, our children are bulk billed* (Regional centre participant 011). The cost of health services influenced treatment options, with some participants preferring primary care: *Their GP is bulk billed, which is good, whereas their paediatrician is about $200 a visit* (Regional centre participant 011).

Participants from the remote communities faced interconnected indirect costs: *You do have the cost of travelling that distance from home with the increased price of fuel and just the general wear and tear and such on the vehicle* (Remote community participant 004), leading to challenges when accessing primary care: *Those extra expenses would be a deterrent from doing things that you don't consider absolutely essential, like going to the doctor* (Remote community participant 003).

Despite challenges, participants prioritised primary care provider quality over the cost of services. Regional centre participants noted: *I don't have a problem paying for that because this [GP] is very good* (Regional centre participant 014). Small rural town participants also noted this: *I don't mind paying the money because he's a good doctor. I don't want to go to someone that I don't feel comfortable with and it's bulk billed, and I just don't get the results that I want* (Small rural town participant 002).

### 3.4 Timeliness

A patient access hierarchy was evident in the regional centre where wait times for primary care appointments were shorter for participants better‐known to their clinic: *I can ring and at short notice they will make it happen … I've been lots of times and they just fit around me* (Regional centre participant 015). Certain small rural town participants also noted this: *I ring up and say I need to see the doctor and I say it's [participant] here, they know me and they book me in as soon as they can* (Small rural town participant 008).

Participants from the regional centre without regular primary care appointments faced delays: *I do find though that if I ring up to make an appointment it is either a week or 2 weeks wait* (Regional centre participant 005). This was also noted for participants in the small rural town: *If you're making an appointment with them … you've got to book 2–3 weeks ahead* (Small rural town participant 005).

Primary care access delays created challenges across all levels of rurality. Regional centre participants experienced missed opportunities which sometimes resolved without medical intervention: *If you can't get in to see a doctor, well then, you've just got to wait … usually by the time you get to the doctor … I think I'm nearly over it now because I needed to see you 3 weeks ago* (Regional centre participant 003).

Small rural town participants faced challenges with medication management due to extended wait times to access primary care: *I went from being able to book within a couple of days … to having to wait 4 weeks … I didn't really monitor my medication that much* (Small rural town participant 002).

Participants from the remote communities experienced the most prominent delays when accessing primary care: *A 2 week wait, 45 min to get to the doctor* (Remote community participant 001), leading to an assumption of the condition's self‐resolution: *If I have to wait 3 weeks to get in, my back is going to be better by then, so I'll just keep taking Panadol* (Remote community participant 003).

### 3.5 Accommodation

Primary care services in the regional centre demonstrated a positive approach to accommodation through digital booking systems: *I actually book my appointments online … I can book weeks and weeks and weeks in advance* (Regional centre participant 006), though technological advancements challenged older participants: *I'm a bit of a digital dinosaur … whether it's smart phones, I'd rather throw the … thing away half the time* (Regional centre participant 001).

In the small rural town, primary care services achieved accommodation needs through service integration: *We can generally get most of the things that we need to go to a GP for done there. Our GP clinic has got a nurse, so that means that we have the kids' vaccines* (Small rural town participant 003).

Primary care services in the remote communities showed flexibility with accommodation of health needs through a certain level of provider understanding of the remote community context: *Some of the GPs that we've had in our community understand that we live in a [remote] area and might not be able to readily travel further for more medical assistance* (Remote community participant 001).

### 3.6 Acceptability

Acceptability operated within the level of primary care need and varied by the level of rurality. Many participants prioritised competent primary care providers over cultural alignment: *I don't mind if it's a Chinese doctor, Indian doctor, I don't care as long as they're trained* (Regional centre participant 003).

Small rural town participants demonstrated acceptability through cultural connections with primary care providers: *O* *ur cultures are very similar … it's really good to talk to someone who knows what wavelength I'm on* (Small rural town participant 009).

Participants from the remote communities put primary care access needs over cultural preferences: *I don't really have a view either way. I don't have any particular social or cultural beliefs … around here is pretty open for everyone* (Remote community participant 002).

### 3.7 Awareness

Established primary care provider relationships emerged as important to primary care access in the regional centre and small rural town but were absent in the remote communities. Continuity of care enabled efficient and comprehensive primary care: *The main reason why I like to try and stick to the same GP is that you have a continuity of information flow and understanding [awareness] of what your issues are* (Regional centre participant 003). Primary care provider relationships were so valued that participants refused to access alternative primary care providers: *We've been going to him for at least 20 odd years so, if he's not there and there's another doctor to see, we refuse to see the other doctors* (Regional centre participant 005).

Small rural town participants also valued communication and engagement with primary care providers: *It's good to have a doctor who will ask. It makes me actually talk to her about problems because I've always had problems before about talking to doctors* (Small rural town participant 009).

Fragmented primary care created increasing challenges across all levels of rurality. Regional centre participants experienced rushed consultations: *She was very short with me because I'm not her normal patient … I feel like they try to brush you off and try to rush you through* (Regional centre participant 006).

Small rural town participants faced increasing primary care provider changes: *Every time it's been a different doctor … when you walk through the door you're a brand new face to them* (Small rural town participant 006).

The need for locums in the remote communities led to challenges when accessing primary care: *The transient nature of the doctors in our community means that you might not see the same doctor that you saw last time, so you kind of aren't building that relationship* (Remote community participant 001).

### 3.8 Geographic Fragility

An additional theme emerged in the remote communities termed geographic fragility, whereby single events within the community fundamentally disrupted access to primary care (e.g., provider departure, service closure, or transport disruptions). This did not strongly align with any of the existing seven dimensions. Remote community participants were needing to weigh up whether their health needs justified the challenge(s) of accessing primary care. This uncertainty forced stressful decision making: Y*ou're not sure if the doctor is going to treat you. You're not sure if you're actually going to get a result … so it's not really worth going all that way* (Remote community participant 001).

Remote participants and residents also assessed the quality of primary care providers before investing in extensive travel to access primary care: *If we know there's no doctor in town or we've heard bad reports about them … then we're not going to spend the time in the car* (Remote community participant 001).

Additionally, access to primary care required integration into routine life planning: *You have to factor in your medical visit in association with when you are going to town next* (Remote community participant 004).

Geographic fragility created remote community anxiety: *My biggest concern is whether we will continue to have that service at all because I look at other towns in this area … that haven't had doctors* (Remote community participant 002). However, participants showed gratitude for the limited primary care services available within their community: *We are just grateful that we actually have a doctor in town at all* (Remote community participant 002).

### 3.9 The Interconnected Nature of Access Dimensions

This study demonstrates how availability serves as a foundational dimension of access that influences all other dimensions. When primary care providers leave a rural or remote community, this in effect impacts all other dimensions, for example, geography (increased travel distances to alternative providers), affordability (higher costs associated with seeking care in a nearby community), timeliness (longer wait times to visit alternative providers), and awareness (repeating health history to new providers). This interconnectedness suggests that rural and remote communities exhibit complexities when accessing primary care that cannot be addressed by examining individual dimensions in isolation.

### 3.10 Why the Level of Rurality Matters

Primary care access operates as a multifaceted system where dimensions of access interconnect, in addition to varying with the level of rurality. Regional centre residents faced moderate, manageable challenges with alternative primary care pathways available. Small rural town residents experienced more prominent challenges with limited primary care alternatives. Residents of the remote communities faced the most complex challenges across all dimensions when accessing primary care, as well as geographic fragility, whereby a single change in circumstances requires extensive adaptation strategies. These findings demonstrate that effective primary care interventions must address the multifaceted and interconnected nature of primary care access rather than targeting dimensions in isolation, with particular attention to the geographic fragility that characterises primary care access in remote and very remote communities.

## 4 Discussion

This study provides the first comprehensive exploration of how the seven dimensions of access from the Russell framework [20] vary across different levels of rurality. The research identifies that residents in regional, rural, and remote Australian communities face multiple challenges when accessing primary care. Access to primary care operates as a complex, interconnected system where the dimensions are shaped by factors including limited healthcare workforce (availability), long wait times for appointments (timeliness) and fragmented healthcare (awareness). Notably, the impact of each dimension varies somewhat with the level of rurality, indicating a complex relationship between geographic location and primary care access. It is acknowledged that the specific population group recruited for the study (i.e., mostly female participants and those reporting good health) may limit the transferability of findings to other groups or populations with different health needs.

The findings demonstrate that changes in workforce availability create flow‐on effects across dimensions of access including geography, affordability, timeliness, and awareness. These findings challenge traditional models and frameworks that address certain dimensions of access in isolation [19, 26], suggesting that effective interventions must acknowledge the interconnected factors that influence access to primary care. The research identified a new theme, termed geographic fragility, most relevant to participants in the remote communities. Geographic fragility evolved around the potentially quick‐changing nature of access for remote community residents, forcing them to weigh up whether their health needs, and those of their family, justified the burden of accessing primary care. This created uncertainty when accessing primary care, often forcing residents to make concerning choices about their health needs that change primary care access behaviour into a complex decision‐making process.

The moderately increasing intensity of primary care access challenges across different levels of rurality, identified in this study, supports previous findings by McGrail and colleagues regarding geographic gradients in healthcare access [27], but extends this understanding by demonstrating how primary care access forms a multidimensional system that intensifies with the level of rurality.

While our finding suggests participants prioritise quality (awareness) over cost (affordability), it is recognised that this relationship is complex. Some participants indicated that the timing of primary care visits could be influenced by pay cycles, suggesting that while cost may not be a primary enabler, financial limitations can still affect primary care access behaviour. Consequently, this research contradicts assumptions about affordability being a priority when accessing primary care [28, 29, 30, 31].

This research suggests that residents in rural and remote communities value ongoing primary care provider relationships and care continuity. The crucial role that primary care providers play in continuity of care across all levels of rurality supports rural and remote workforce strategies that focus on recruitment, community integration and retention [32, 33, 34]. The findings indicate that investing in long‐term primary care provider relationships offers benefits across multiple access dimensions simultaneously, potentially enabling a more efficient use of resources, as opposed to addressing dimensions in isolation.

An interesting finding within this study was regarding access hierarchies where some participants who utilised primary care more frequently appeared to bypass certain access challenges, such as being more likely to avoid extensive waiting times for appointments. This finding somewhat challenges equity principles in healthcare delivery [35]. While established participants receive preferential access to address their ongoing (chronic) needs, newcomers and occasional users face timeliness challenges regardless of need. This pattern was observed for regional centre and small rural town residents and may suggest that healthcare services inadvertently create access inequities based on utilisation patterns rather than need.

Our identification of geographic fragility as a new theme extends previous rural health research that has recognised workforce turnover and retention challenges in remote communities [36], and identified long‐term supply sustainability issues [37]. This is in addition to research that has identified the most important factors associated with rural and remote primary care provider turnover [38]. The recognition of geographic fragility has important theoretical implications for healthcare access. Traditional access models and frameworks, derived from metropolitan context, that often assume continuation of health services or consistent healthcare availability [16, 18, 39], may inadequately address access experiences in remote communities, where increased rurality and low primary care provider retention contribute to uncertainty and vulnerability among residents. This study reveals how geographic fragility creates complex primary care access challenges for remote community residents by creating uncertainty and community‐level vulnerability.

The adaptation strategies developed by residents in the remote communities suggest that healthcare systems should recognise and support place‐based approaches rather than replacing them with urban‐derived access models or frameworks [16, 17]. The residents that assess primary care provider quality, the integration of primary care access into broader life planning, and the development of primary care provider relationships represent innovations that could inform healthcare policy.

The lack of community cultural understanding among metropolitan‐trained and internationally trained primary care providers highlights the need for rural‐specific awareness. The findings align with research by Farmer and colleagues on rural culture [40], while revealing how these relationships become increasingly important as the level of rurality increases and alternative points of access reduce. This awareness should extend beyond clinical skills to include a deeper understanding of geographic limitations, cultural values, and the practical realities of living in remote and very remote communities. Interventions should aim to develop primary care practices that recognise both the challenges and strengths of smaller rural and remote communities.

From a policy perspective, this research suggests that focusing on single dimensions may have limited success in improving access to primary care across different levels of rurality. For instance, increasing bulk billing rates (affordability) without improving primary care provider recruitment and retention (availability) might offer short‐term solutions without fixing long‐term access challenges. Likewise, improving transportation (geography) without ensuring primary care providers are educated in the cultural context of rural and remote communities (acceptability) could increase utilisation but not necessarily improve health outcomes.

### 4.1 Limitations and Strengths

Several limitations should be recognised, particularly the low number of participants in the remote and very remote communities, which may make the transferability of findings more difficult. Since the study was restricted to Queensland, Australia, its findings may have limited applicability to other states and territories. Future research should explore dimensions of access to primary care in other rural and remote communities and states. This study's strengths include its use of the Russell framework, offering insight into how rural and remote primary care access challenges are interconnected and prioritised according to their importance. The identification of a new theme is an important theoretical contribution that emerged from the analysis. Using the Modified Monash Model to categorise rurality levels ensured consistency with Australian healthcare policy.

## 5 Conclusion

This research reveals that regional, rural, and remote Australian communities face multiple challenges when accessing primary care across different levels of rurality. The findings question traditional access frameworks where evidence largely derives from metropolitan contexts, proposing instead that effective solutions must recognise the multifaceted and interconnected nature of primary care access and address the links between different dimensions. Highlighting geographic fragility as a new theme relevant to the remote and very remote communities emphasises the uncertainty and vulnerability that these residents face when accessing primary care. Importantly, this research has demonstrated that improving access to primary care in regional, rural, and remote communities requires more than increased primary care service availability. It involves establishing a healthcare system that is reliable, appropriate for the culture of the local community, and responsive to the specific contexts of rural and remote communities.

## Author Contributions

**Maddie Higgins:** conceptualisation; writing – original draft (lead); formal analysis; writing – review and editing. **Matthew McGrail:** formal analysis; writing – review and editing. **Tiana Gurney:** formal analysis; writing – review and editing.

## Ethics Statement

A low‐risk ethics application was approved by the University of Queensland Human Research Ethics Committee (2022/HE000199).

## Data Availability

The data that support the findings of this study are available from the corresponding author upon reasonable request.
